# Anatomical Variation of Absent Facial Vein: Implications for Facial Reanimation Surgery

**DOI:** 10.1002/hed.70274

**Published:** 2026-04-08

**Authors:** Cam T. Nguyen, Mark Fricke, Justus Osterloh, Ayla A. Hohenstein, Steffen U. Eisenhardt

**Affiliations:** ^1^ Medical Center and Faculty of Medicine—University of Freiburg Department of Plastic and Hand Surgery Freiburg Germany

**Keywords:** facial reanimation surgery, facial vascular anatomical variation, facial vein aplasia

## Abstract

**Background:**

The facial vein is the standard recipient vessel in facial reanimation surgery. Its complete absence is rarely described but may cause major challenges during free functional muscle transfer (FFMT). This study aimed to determine the prevalence and predictors of facial vein absence and assess its surgical relevance in facial reanimation patients.

**Methods:**

A retrospective analysis of 198 patients who underwent FFMT between 2005 and 2025 was performed. Facial vein presence was evaluated intraoperatively, and all patients had preoperative Doppler ultrasonography. Potential predictors were analyzed using logistic regression and Fisher's exact test.

**Results:**

Facial vein aplasia occurred in 12 patients. Aplasia was significantly associated with congenital etiology and syndromic palsy, especially Moebius syndrome. No associations with age, sex, operative time, or complications were found.

**Conclusions:**

Facial vein aplasia is strongly linked to congenital and syndromic palsy. Preoperative Doppler ultrasonography is recommended for identifying venous anomalies and optimizing surgical planning.

**Trial Registration:**

Freiburger Register Klinischer Studien (FRKS): Number FRKS005811

## 1 Introduction

The facial vein is an important component of the venous drainage system in the facial region. Anatomical studies have demonstrated considerable inter‐individual variability in both the location and course of the facial vein, with occasional reports of complete aplasia [1, 2, 3, 4, 5]. The absence of the facial vein represents a significant challenge in facial reanimation surgery, particularly in patients undergoing free functional muscle transfer (FFMT), as it necessitates the identification and utilization of alternative venous drainage pathways. This anatomical variation can substantially influence surgical planning and outcomes. Despite its clinical significance, there is a notable paucity of research regarding the prevalence and surgical implications of facial vein absence in the context of facial nerve palsy. Furthermore, existing data are limited to small case series, restricting the generalizability of current knowledge [6, 7].

Facial reanimation surgery has progressively evolved to optimize both aesthetic and functional outcomes for patients with facial paralysis, encompassing a range of techniques [8, 9]. The development of FFMT using the gracilis muscle marked a major advancement, providing predictable dynamic motion through reliable reinnervation [10]. With subsequent refinements, particularly through the combination of static and dynamic techniques, surgical strategies have further advanced to enhance symmetry [11, 12]. Within this framework, the success of the operation relies critically on ensuring adequate perfusion of the transplanted muscle, which in turn depends on the identification and safe preparation of suitable recipient vessels. The absence of the facial vein may therefore represent a key intraoperative challenge.

Consequently, there is an unmet need for systematic investigation of this anatomical variation. The objective of this study is to determine the prevalence of facial vein absence in a large patient cohort and to assess its association with specific risk factors, including age, etiology of facial nerve palsy, and the presence of congenital syndromes such as Moebius syndrome or CHARGE syndrome. It is anticipated that these findings will enhance preoperative diagnostic protocols and surgical decision‐making, thereby reducing the risk of complications associated with unrecognized venous anomalies.

## 2 Materials and Methods

This retrospective cohort study was carried out at the Department of Plastic and Hand Surgery, Medical Center—University of Freiburg, Germany. The study was approved by the Ethics Committee of the Albert Ludwig University of Freiburg (approval number 25‐1200‐S1‐retro).

All patients who underwent FFMT for facial reanimation between July 2005 and March 2025 were screened for eligibility. Patients were included if an intraoperative dissection of the facial vein was performed. Individuals were excluded if the facial vein was not explored due to planned use of alternative recipient vessels, such as the temporal artery or vein, or if a history of previous surgery or trauma to the affected hemiface was present.

Data were collected retrospectively via the department's electronic patient records, which document preoperative diagnosis, intraoperative findings, recipient vessels used, and relevant patient information. The following variables were extracted for analysis: patient sex, age, affected side, etiology of facial palsy (isolated congenital, syndromic congenital, or acquired), intraoperative findings of the recipient vessels, operation time, and perioperative complications. All surgical procedures as well as preoperative assessments were performed by a single senior surgeon (S.U.E.) with specialization in facial reanimation surgery, corresponding to level five of surgical experience [13]. For patients with bilateral congenital facial palsy, the absence of the facial vein on one or both sides were considered as facial vessel aplasia. All patients were routinely evaluated preoperatively by Doppler ultrasonography to confirm the presence or absence of the facial artery and vein, as well as to assess the transverse facial vein as a potential alternative recipient vessel. These findings were systematically correlated with intraoperative observations to assess the diagnostic accuracy of vascular mapping for recipient vessel selection.

**FIGURE 1:**
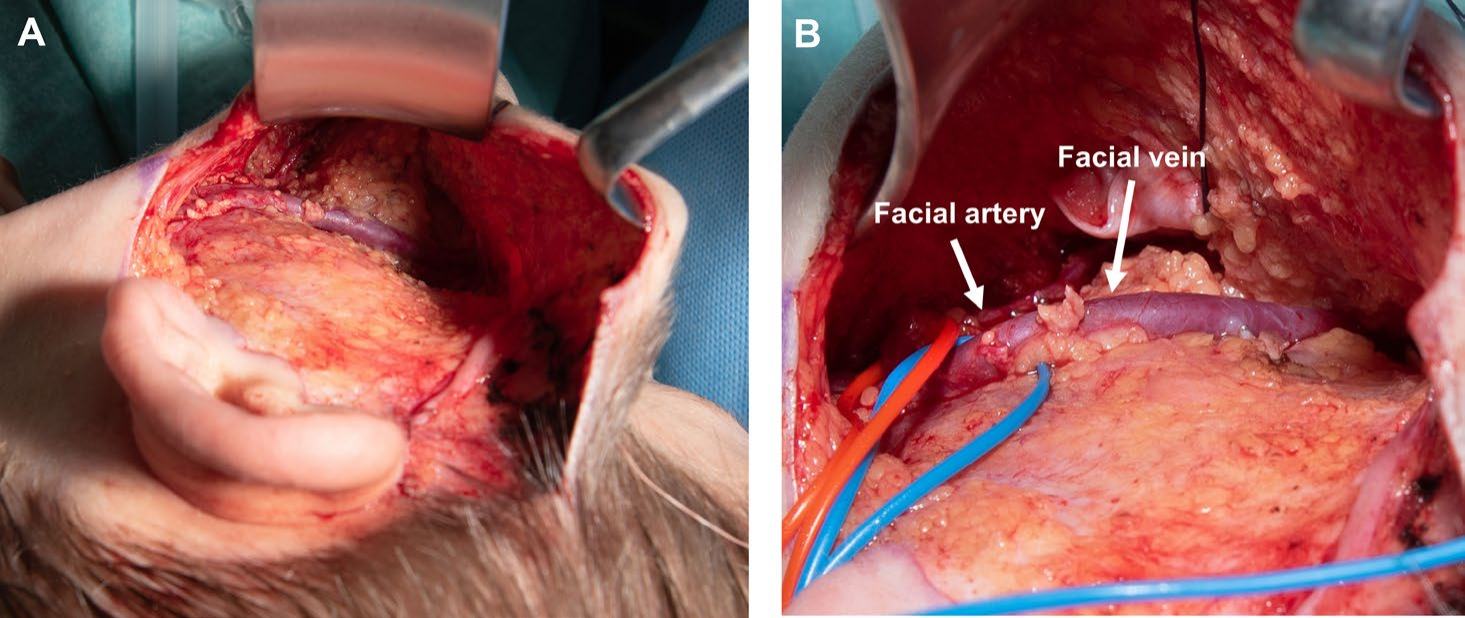
Typical anatomy via preauricular approach demonstrating a prominent facial vein (A). The facial vein is indicated by a blue vessel loop, and the facial artery by a red vessel loop (B). [Color figure can be viewed at wileyonlinelibrary.com]

**FIGURE 2:**
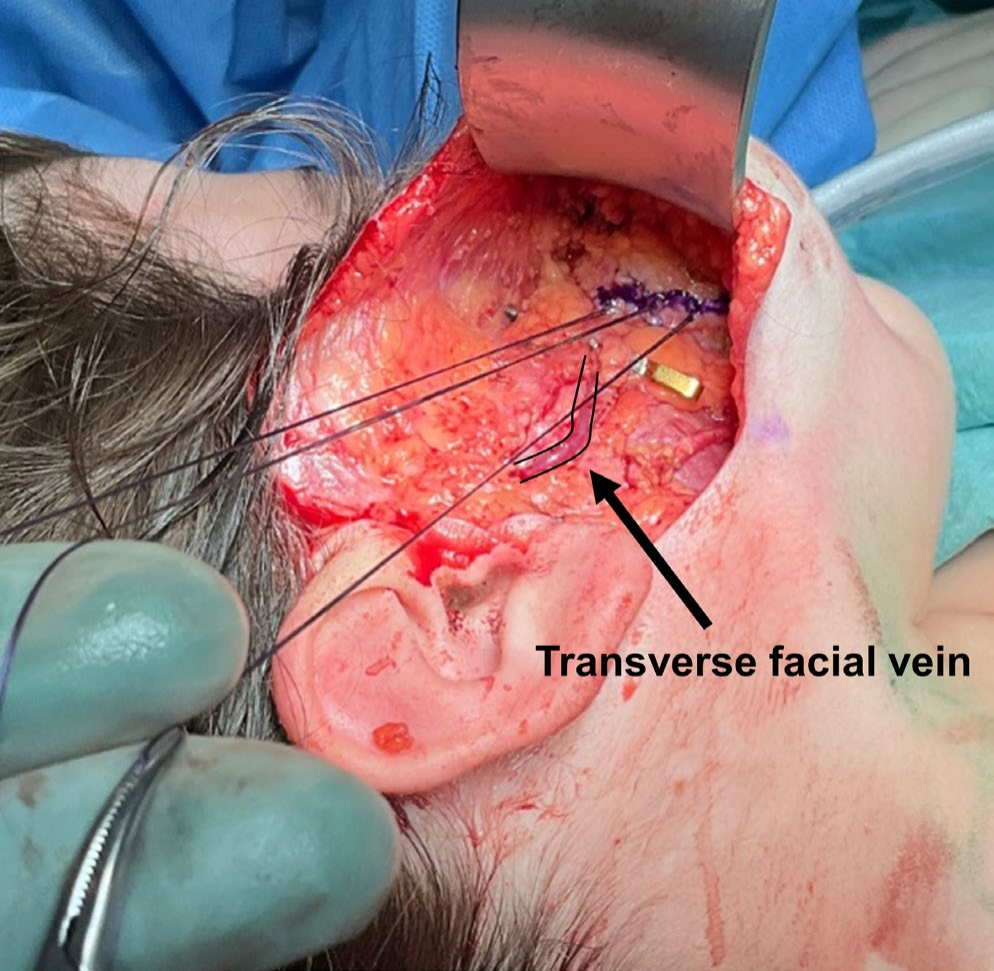
Absence of the facial vein with a prominent transverse facial vein serving as an alternative recipient vessel. [Color figure can be viewed at wileyonlinelibrary.com]

**FIGURE 3:**
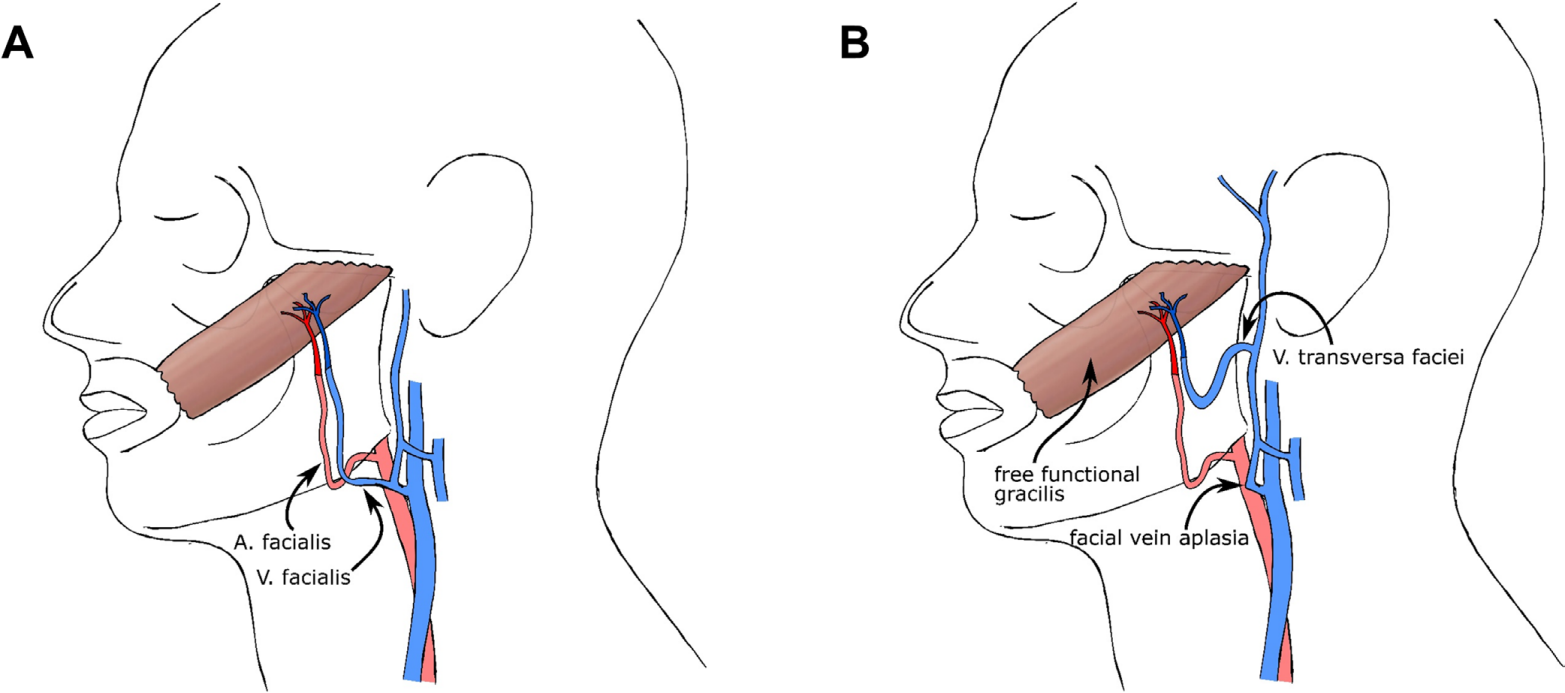
Schematic illustration of gracilis muscle anastomosis to the facial artery and vein (A), and to the transverse facial vein in case of facial vein aplasia (B). [Color figure can be viewed at wileyonlinelibrary.com]

The FFMT technique performed in this study has been previously published in our clinic [11, 14, 15]. The intraoperative procedure involved a modified facelift incision, followed by sub‐SMAS dissection to expose and mobilize the facial artery and vein, which were clipped and reflected preauricularly for subsequent microvascular anastomosis (Figure 1). All anastomoses were performed using microsutures. The facial artery served as the recipient artery in all reconstructions. In cases where the facial vein could not be identified, the transverse facial vein was sought as an alternative recipient vessel (Figure 2). In all these cases, the transverse facial vein showed a significantly increased caliber when compared to patients with facial vein. This increased caliber was matching the venous vessels of the gracilis muscle. Schematic illustrations demonstrate the anastomosis of the gracilis muscle to the facial artery and vein and anastomosis to the transverse facial vein in case of facial vein aplasia (Figure 3). Venous outflow was continuously monitored postoperatively by implantation of a Cook Doppler probe on the recipient vein, which was maintained for 7 days in accordance with the manufacturer's recommendations (Cook Medical Europe Ltd., Limerick, Ireland) [16].

The primary objective was to identify predictors for congenital absence of the facial vein. Potential sources of bias included the retrospective design with potential selection bias. To address confounding, all patients were systematically evaluated preoperatively with Doppler ultrasonography, ensuring comparable assessment for vessel presence across groups. Cases with missing critical data were not present in this dataset.

## 3 Statistical Analysis

Statistical analysis was performed using GraphPad Prism (version 10.6, GraphPad Software Inc., Boston, MA, USA). Descriptive statistics are presented as median and interquartile range (IQR) for non‐normally distributed data and as mean with standard deviation (SD) for normally distributed data. Normality of continuous variables was assessed using the Shapiro–Wilk test. Associations between the absence of the facial vein and categorical predictors were assessed using Fisher's exact test, as appropriate. Odds ratios (ORs) with corresponding 95% confidence intervals (CIs) were calculated to quantify the strength of association between each predictor and facial vein absence. The relationship between age and absence of the facial vein was analyzed using logistic regression, with age as a continuous variable and facial vein presence as the binary outcome. All statistical analyses were two‐tailed, and a *p* value < 0.05 was considered statistically significant.

## 4 Results

Between July 2005 and March 2025, a total of 222 free functional gracilis muscle transfers for facial reanimation were performed on 214 patients at our clinic. Of these, 198 patients met the inclusion criteria. Sixteen patients were excluded because intraoperative dissection of the facial vein was not performed due to the planned use of alternative recipient vessels or because the patients had a history of previous surgery or trauma to the affected hemiface.

The final study cohort consisted of 120 female (61%) and 78 male (39%) patients. The median age at the time of surgery was 38 years (IQR 19.75–57; range 5–73 years), comprising 44 (22%) pediatric and 154 (78%) adult patients. Among the 198 included patients, 99 presented with right‐sided unilateral facial paralysis, 87 with left‐sided paralysis, and 12 with bilateral facial paralysis. In patients with bilateral paralysis, the vascular anatomy was symmetrical, with the facial vein either present or absent on both sides. Intraoperative exploration revealed that 12 cases (6.1%) showed an absence of the facial vein. Among those, the transverse facial vein was used as the alternative recipient vein in all cases. In our cohort, the congenital absence of the facial vein was not accompanied by an absence of the facial artery. The facial artery was used as the recipient artery in all reconstructions.

Preoperative Doppler ultrasonography findings correlated closely with intraoperative vascular anatomy. In 194 of 198 patients, Doppler sonography correctly identified the presence of a facial vein, yielding a sensitivity of 98%. Among the 12 cases with congenital facial vein absence, 9 were accurately predicted preoperatively, corresponding to a specificity of 75%. The positive predictive value was 98.5%, confirming the high reliability of a positive Doppler finding, while the negative predictive value was 69.2%, indicating that a negative result does not definitively exclude the presence of a facial vein. Overall diagnostic accuracy for facial vein detection in our cohort was 97.1%.

The etiology of facial palsy was congenital in 44 patients (22%) and acquired in 154 patients (78%). Among patients with congenital facial palsy, six patients had Moebius syndrome, four had CHARGE syndrome, and one had another unspecified congenital syndrome with craniofacial dysmorphia including cleft palate and complex congenital heart defect. Patient demographics are detailed in Table 1, and a comparison of patients with and without facial vein is summarized in Table 2. No perioperative complications, such as hematoma, arterial or venous thrombosis (immediate or delayed), flap loss or infection, were observed in any patients with congenital absence of the facial vein. The mean operative time did not differ significantly between groups, with patients with a facial vein averaging 306.4 ± 5.23 min and 304.42 ± 18.07 min for those without (*p* = 0.8484).

**TABLE 1:** Patient demographic and clinical characteristics (*n* = 198).

Characteristics	*n* (%)
Sex	
Female	120 (61)
Male	78 (39)
Age (in years)	
Median (IQR; range)	38 (19.75–57; 5–73)
Pediatric patients	44 (22)
Adult patients	154 (78)
Affected side	
Right	99 (50)
Left	87 (44)
Both	12 (6)
Etiology	
Congential	44 (22)
Moebius syndrome	6 (3)
CHARGE syndrome	4 (2)
Other congenital syndrome	1 (0.5)
Acquired	154 (78)
Aplasia of facial vein	12 (6.1)
Perioperative complications	0 (0)

**TABLE 2:** Comparison of patient characteristics between patients with and without facial vein (*n* = 198).

	Absent facial vein (*n* = 12)	Present facial vein (*n* = 186)
Congenital facial palsy	8 (66.7%)	36 (19.4%)
Acquired facial palsy	4 (33.3%)	150 (80.6%)
Syndromes	5 (41.7%)	7 (3.8%)
Moebius	3 (25%)	3 (1.6%)
CHARGE	1 (8.3%)	3 (1.6%)
Other	1 (8.3%)	1 (0.5%)
Sex ratio (male: female)	1:1	1:1.6
Age < 18 years	4 (33.3)	40 (21.5%)

**FIGURE 4:**
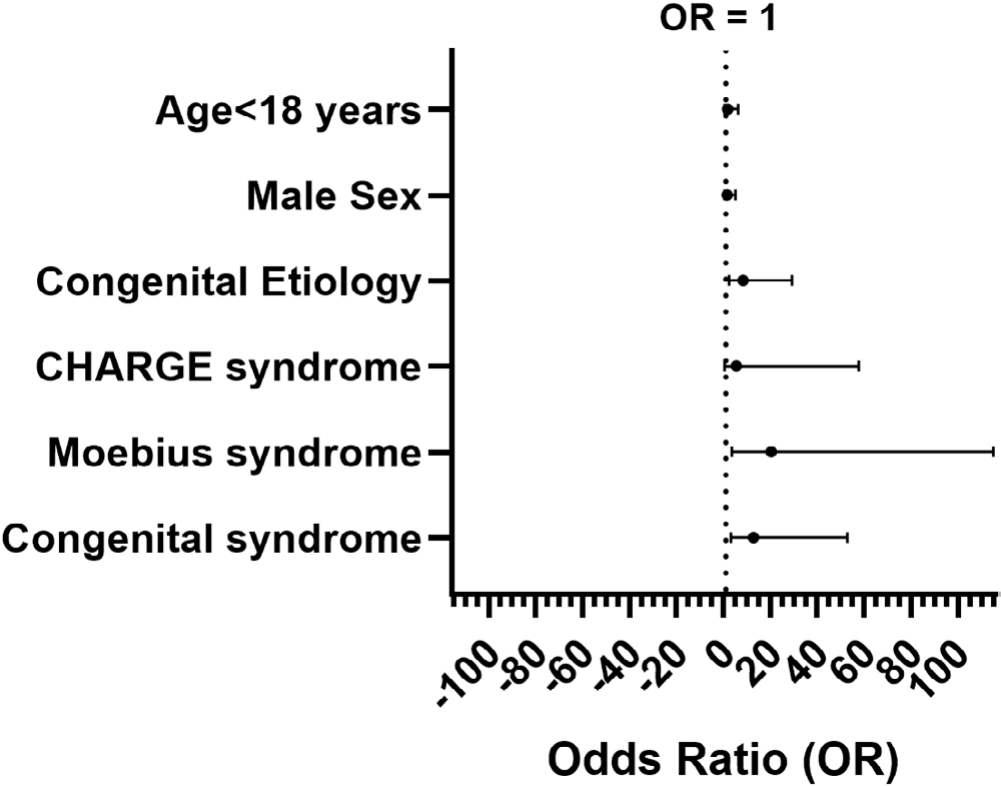
Forest plot illustrating the odds ratios and 95% confidence intervals of potential predictors associated with the absence of the facial vein.

Further analysis revealed that absence of the facial vein was present in 36.4% of those with any congenital syndrome, in 50% of patients with Moebius syndrome, and in 25% of those with CHARGE syndrome. Patients with syndromic facial palsy were significantly more likely to have an absent facial vein compared to those without a syndrome (*p* = 0.0021; OR 12.79, 95% CI: 3.097–52.792). In subgroup analysis, this association was particularly pronounced in patients with Moebius syndrome (*p* = 0.0031; OR 20.333, 95% CI: 3.588–115.224), whereas the association between CHARGE syndrome and absent facial vein did not reach statistical significance (*p* = 0.2228; OR 5.545, 95% CI: 0.532–57.773). A congenital non‐syndromic cause of facial palsy in general was also strongly associated with facial vein absence (*p* = 0.0008; OR 8.333, 95% CI: 2.378–29.207). No significant associations were found for male sex (*p* = 0.545; OR 1.583, 95% CI: 0.492–5.099) or age below 18 years (*p* = 0.471; OR 1.825, 95% CI: 0.523–6.371). A forest plot illustrating the odds ratios of different predictors for facial vein absence is presented in Figure 4.

## 5 Discussion

This study investigated the prevalence and clinical significance of congenital facial vein absence in patients undergoing FFMT for facial reanimation. In a cohort of 198 patients, congenital absence of the facial vein was identified intraoperatively in 6.1% of cases. Facial vein aplasia was significantly associated with congenital etiology and particularly syndromic forms of facial palsy. The highest rate was observed in patients with Moebius syndrome (50%), while CHARGE syndrome demonstrated a trend towards an increased risk (OR 5.545) but did not reach statistical significance due to the small sample size. No association was found between facial vein absence and sex or pediatric status.

These findings are highly relevant for facial reanimation surgery, since the success of FFMT in restoring dynamic function depends on reliable vascular anastomosis. The facial artery and vein are the preferred options due to their proximity to the modiolus, predictable caliber, and favorable handling characteristics, facilitating optimal perfusion and drainage of the transferred muscle [6]. Our findings were corroborated by previous anatomical and surgical studies, which showed that, although the course of the facial vein is typically consistent, rare cases of aplasia do occur, especially among patients with facial palsy (Table 3).

**TABLE 3:** Summary of studies investigating facial vein anatomy and absence.

Author and year	Diagnostic tool	No. patients	Facial palsy	No. facial vein absences
Bondaz et al. (2014) [1]	CT angiography	330	No	0
Wang et al. (2022) [2]	CT angiography	150	No	0
Cotofana et al. (2017) [3]	Anatomic dissection cadaver	72	No	0
Lohn et al. (2011) [4]	Anatomic dissection cadaver	197	No	2
Renshaw et al. (2007) [5]	Color doppler	200	No	1
Henry et al. (2014) [6]	Intraoperative dissection	87	Yes (53 congenital)	15
Butler et al. (2014) [7]	Intraoperative dissection	47	Yes (47 congenital)	8
Nguyen et al.	Intraoperative dissection	198	Yes (44 congenital)	12

Two studies with 330 and 150 patients utilizing CT angiography among healthy populations reported no single case of facial vein absence [1, 2]. Similarly, Renshaw et al. [5] used color Doppler ultrasonography in a cohort of 200 non‐palsy patients and found just one case of absence. Two separate cadaveric studies were conducted, comprising 197 and 72 specimens, respectively. Only one of these studies reported the absence of the facial vein, identifying two cases out of 197 cadavers [3, 4]. These findings contrast sharply with the markedly higher prevalence observed in our cohort, and analysis of facial palsy populations elucidates this discrepancy. Henry et al. [6], for instance, documented 15 cases of facial vein absence in 87 patients, of which 53 patients had congenital facial palsy, while Butler et al. [7] identified 8 absent facial veins in a cohort of 47 congenital facial palsy patients, noting a predominance among syndromic cases. Within our cohort of 198 patients, of whom 44 patients presented with congenital facial palsy, 12 patients lacked a facial vein.

The consequences of facial vein aplasia during reconstructive surgery are considerable. When the facial vein is absent, alternative recipient veins must be selected intraoperatively. Our approach has favored the transverse facial vein, which we found reliably prominent in patients with facial vein aplasia and is accessible given the gracilis muscle's generous vascular pedicle length. The increased risk of facial vein aplasia in specific patient cohorts necessitates thorough preoperative vascular assessment, such as with Doppler ultrasonography or CT angiography [1, 2, 5, 17], to identify suitable recipient vessels and avoid intraoperative surprises. Nevertheless, we do not generally advocate CT angiography because of radiation exposure and would restrict its use to selected complex cases, such as patients with multiple prior operations, trauma, or suspected major vascular anomalies, in whom detailed three‐dimensional vascular information is essential for planning and cannot be obtained adequately by ultrasound alone. Crucially, our protocol of preoperative Doppler assessment enabled us to anticipate such variations and adapt our surgical plan accordingly, resulting in no increase in operative time or complication rates among affected patients. This finding stands in contrast to reports where a lack of vascular mapping necessitated modification of the muscle flap. Butler et al. [7] reported that they use a segmental latissimus dorsi muscle flap in syndromic cases instead of their standard pectoralis minor muscle to provide a neurovascular pedicle of sufficient length to reach the facial vessels and have flexibility to reach another recipient vessel when facial vessels are absent [18]. Additionally, our routine use of an implantable Doppler probe for postoperative vascular surveillance enables early detection of venous or arterial thrombosis, contributing to high flap survival [16].

The embryological basis for facial vein absence centers on the remodeling of the craniofacial venous plexus during development. The facial vein arises from the anterior part of this plexus, with connections to the primitive linguofacial vein and the developing internal and external jugular veins. Aberrant regression or persistence of anastomotic channels may produce variable drainage patterns or complete vein aplasia. A phenomenon seen more commonly in syndromic craniofacial disorders [3, 19, 20, 21]. This is exemplified by Huntsman's 1992 report of 3 out of 9 patients with hemifacial microsomia exhibiting absent facial vessels [22], emphasizing the clinical importance of understanding these embryological mechanisms.

Strengths of this study include the large cohort size and the complete dataset without missing values. The main limitation is its retrospective design. Nonetheless, the results offer valuable guidance for preoperative planning and intraoperative decision‐making in facial reanimation surgery. These findings strongly support the routine use of preoperative vascular mapping with Doppler ultrasonography for all patients, with particular attention to those with congenital or syndromic facial palsy. Further research is warranted to refine imaging protocols and to explore the embryological basis of these vascular anomalies in syndromic facial palsy.

In conclusion, facial vein aplasia is significantly more common among congenital facial palsy patients, especially those with syndromic forms. Preoperative vessel assessment using Doppler ultrasonography is essential for efficient and safe intraoperative selection of recipient veins.

## Author Contributions

C.T.N. and S.U.E. conceived the study. All authors participated in the acquisition, analysis, and interpretation of the data. C.T.N. drafted the article, and all authors revised it critically for important intellectual content. All authors gave final approval of the version to be published and agreed to be accountable for all aspects of the work.

## Funding

The authors have nothing to report.

## Ethics Statement

Ethical approval for this study was obtained from the Ethics Committee of the Albert Ludwig University of Freiburg (approval number 25‐1200‐S1‐retro).

## Consent

The requirement for informed consent was waived due to the retrospective nature of the study.

## Conflicts of Interest

The authors declare no conflicts of interest.

## Data Availability

The data that support the findings of this study are available from the corresponding author upon reasonable request.
